# Improved Antibiotic Prescribing for Acute Conjunctivitis After Operational Research: A Before-and-After Study in a Ghanaian Eye Hospital

**DOI:** 10.3390/tropicalmed10110301

**Published:** 2025-10-22

**Authors:** Henry Kissinger Ansong, Divya Nair, Joana Abokoma Koomson, Obed Kwabena Offe Amponsah, Jane Frances Acquah, James Buckman, Andrew Ramsay, Paa Kwesi Fynn Hope

**Affiliations:** 1 Bishop Ackon Memorial Christian Eye Centre, Cape Coast P.O. Box AD 184, Ghana; jkoomson1234@gmail.com (J.A.K.); bishopsjane@gmail.com (J.F.A.); james.upspgh@gmail.com (J.B.); pkhhope@gmail.com (P.K.F.H.); 2 Independent Researcher, Scottsdale, AZ 85259, USA; divsnair08@gmail.com; 3 Department of Pharmacy Practice, Kwame Nkrumah University of Science and Technology, Kumasi 00233, Ghana; okoamponsah@knust.edu.gh; 4 School of Medicine, University of St Andrews, St Andrews KY16 9TF, UK; andy.ramsay@st-andrews.ac.uk

**Keywords:** SORT IT, operational research, antimicrobial resistance, conjunctivitis, antibiotics, ophthalmic prescribing, AWaRe classification, antibiotic stewardship, West Africa

## Abstract

Empirical antibiotic treatment is common in acute conjunctivitis despite most cases being non-infectious or viral infections. Operational research (OR) at an eye hospital in Ghana (January–December 2021) identified appropriate antibiotic prescription in 71% of cases. Research dissemination and the sensitisation of key stakeholders followed, including communication of findings and implications to hospital prescribers. We conducted this OR covering January–December 2024 to test the hypothesis that the appropriateness of antibiotic prescriptions will improve, and to investigate the types of antibiotics prescribed and their AWaRe classification. There were 220 acute conjunctivitis cases in 2024, comparable to 2021 (201 cases). Antibiotics were prescribed in 67% of cases in 2024 and 55% in 2021 (aOR 2.51, 95% CI: 1.51–4.19, *p* < 0.001). Antibiotic prescription appropriateness was higher in 2024 than in 2021: 87% and 71%, respectively (95% CI for change: 5.99–25.99%, *p* = 0.001). In 2021, only ACCESS and WATCH antibiotics were prescribed. However, 15% of prescriptions in 2024 were RESERVE antibiotics, and multiple antibiotic prescriptions increased from 10% to 22%. This research demonstrates that regular operational research and interventions have the potential to improve antibiotic prescribing in ophthalmic practice in Ghana. It is imperative that the recommendations made by the initial researchers are fully implemented to protect the efficacy of available antibiotics.

## 1. Introduction

Conjunctivitis is an inflammation of the conjunctival tissue of the eye. Patients present with eyes that can be red, swollen, and painful. There is also frequently a mucoid discharge [1,2,3]. The condition may be categorised as acute, chronic, or recurrent based on the onset and nature of presentation [3,4,5]. Acute conjunctivitis (AC) is commonly defined as conjunctivitis with symptoms of less than 3 weeks’ duration [6,7,8]. It can further be categorised as non-infectious or infectious. Acute non-infectious conjunctivitis may be caused by trauma, chemical irritation, or, most commonly, by allergies. Acute infectious conjunctivitis (AIC) has two main subcategories relating to the type of pathogen involved: bacterial AIC or viral AIC [5,6,7,9]. Due to the lack of microbiological testing to differentiate bacterial from viral conjunctivitis, broad-spectrum antibiotics are frequently used to treat any infectious acute conjunctivitis [7,9]. However, bacterial and viral AIC can be distinguished by taking a thorough patient history and meticulous eye examination, and inappropriate antibiotic treatment could be avoided [3,7,10]. A schematic of acute conjunctivitis categories and a diagnostic aid detailing signs and symptoms of allergic, bacterial, and viral conjunctivitis are provided in Supplementary Materials.

Patients with AIC in Ghana may seek care in the community through pharmacies, primary eye care facilities, or referral hospitals. While primary eye care facilities are staffed solely by optometrists and ophthalmic nurses, referral hospitals also have ophthalmologists. The current first-line treatment for bacterial AIC in Ghana is with antibiotic eye drops and/or ointment. Treatment for viral AIC focuses on symptom relief unless there are mitigating circumstances that indicate antibiotic use, such as suspicion of secondary bacterial infection. Allergic (non-infectious) acute conjunctivitis is treated with mast cell stabilisers and antibiotics are not used unless there are similar mitigating circumstances to those of viral AIC. The antibiotics that are approved by the Ghana Standard Treatment Guidelines (STG) for the treatment of bacterial AIC are chloramphenicol 0.5% or tetracycline 1% ointment, and ciprofloxacin eye drops 0.3% [11].

Patients who return with unresolved symptoms of bacterial AIC usually move from chloramphenicol treatment through to treatment with ciprofloxacin (or alternative antibiotics) until the condition resolves. The Ghana STG recommendations are designed for broad use by both general practitioners and eye care professionals. However, deviations may occur for several reasons. In some cases, eye care professionals adapt treatment based on their specialised training and scope of practice, which may go beyond the general recommendations. In other instances, factors such as medicine availability, patient expectations, and prescriber experience can also influence prescribing behaviour.

There is a growing threat of antimicrobial resistance (AMR) globally [12,13,14,15]. In Ghana, antibiotic resistance has been reported by different studies in health and environmental sciences. Bacteria resistant to STG-recommended antibiotics have been isolated in patients with urinary tract infections, as well as drinking water sources and in healthy pigs [16,17,18,19,20]. Antibiotic use was reported to have been sub-optimal by studies in two eye clinics in Ghana [21,22]. It is therefore imperative to ensure that antibiotic prescription is optimal as outlined in Global and National Action Plans on Antimicrobial Use and Resistance to avert the burden of AMR on the health system [23,24,25,26].

Operational research, carried out through the Structured Operational Research and Training Initiative (SORT IT) and based on 2021 data, found the following antibiotic prescribing practices at the Bishop Ackon Memorial Christian Eye Centre (hereafter referred to as BAMCEC) [22]:

1. A total of 29% of patients receiving antibiotics had been prescribed them inappropriately, according to STG recommendations.
2. A total of 56% of patients receiving antibiotics were prescribed antibiotics from the WATCH category of the WHO AWaRe scheme and the remainder prescribed antibiotics from the ACCESS category.

The AWaRe classification contains three groups of antibiotics (ACCESS, WATCH, and RESERVE). The ACCESS category includes antibiotics for the empirical treatment of common infections, which should be available in all healthcare settings. The WATCH category antibiotics have a higher potential for resistance and their use should be limited. The RESERVE category are “last resort” antibiotics and their use should be reserved for special situations with multidrug-resistant bacterial infections where alternative treatments have failed [25,27].

Following the research, the investigators produced information products, including evidence briefs, outlining why and how the research was conducted, the findings, and the implications for policy and practice. These provided the platform for a number of meetings with stakeholders convened between November 2022 and August 2023. The researchers proposed seven recommendations to address concerns identified during the study. Of these, four recommendations have been implemented:

1. To meet and brief the management of BAMCEC on the outcome and implications of the study.
2. To meet and brief the Head of Ghana Eye Care Secretariat on the outcome and implications of the study.
3. To meet and brief BAMCEC prescribers on the outcome and implications of the study.
4. To establish a Local Antibiotic Stewardship Team (LAST).

Three recommendations are yet to be implemented (as of July 2025):

1. To monitor the use of antibiotics in the Eye Clinic by the LAST.
2. To conduct Continuing Professional Development (CPD) training related to antibiotic prescribing through the Drugs and Therapeutic Committee at BAMCEC.
3. Replication of the study in other eye care facilities with the oversight of the Ghana Eye Care Secretariat.

We considered the package of measures implemented above as an intervention. Details of research dissemination and recommendations have been provided in the Supplementary Materials.

This study builds on the earlier operational research project at BAMCEC (Hope et al. [22]), conducted with partial overlap in the author team. This present study represents an impact assessment of prescribing practices following dissemination and sensitisation efforts.

We hypothesised that the operational research results dissemination and sensitisation will have improved the appropriateness of antibiotic prescribing.

This study aimed to assess whether the appropriateness of antibiotic prescribing improved, and the use of WATCH category antibiotics decreased, following the operational research and the associated dissemination and sensitisation activities. We compared antibiotic prescribing patterns for acute conjunctivitis in two time periods, 1 January –31 December 2021 (the period covered by the operational research) and 1 January –31 December 2024 (following research result dissemination and training) in a specialist eye hospital in Cape Coast, Ghana.

The previous operational research only looked at patients who were being prescribed antibiotics and whether that was conducted appropriately. We, in addition, looked at patients with acute conjunctivitis who did NOT receive antibiotics and whether the withholding of antibiotics was appropriate. An attempt was made to collect baseline data on patients not receiving antibiotics in 2021 to be compared with data from 2024. However, not all treatment cards belonging to these patients could be obtained.

## 2. Materials and Methods

### 2.1. Study Design

The study was a retrospective before–after observational study, using routinely available secondary data from an electronic medical record (EMR) system and supplemented with data from patient treatment cards in two time periods: 2021 (before intervention) and 2024 (after intervention).

### 2.2. Settings

#### 2.2.1. General Setting

The study was conducted in Cape Coast, Ghana. Ghana is a West African country that shares borders with Burkina Faso to the north, Togo to the east, Cote d’Ivoire to the west, and the Gulf of Guinea in the south. The population of the country was reported to be 30.8 million in the 2021 population and housing census [28]. There are sixteen administrative regions in Ghana, and Cape Coast is the capital of the central region.

#### 2.2.2. Specific Setting

BAMCEC is a specialist hospital that offers eye care services to a wide range of patients in and around Cape Coast.

Most clients of the facility receive services with their National Health Insurance, while others do so with private health insurance or pay cash to receive care. There are also patients from neighbouring Cote d’Ivoire who attend clinics at BAMCEC regularly. The Centre has tertiary-level eye care diagnostic equipment and a fully functional operating theatre.

The Centre has a staff strength of 54, consisting of one part-time ophthalmologist, five full-time optometrists, four full-time ophthalmic nurses, seven opticians, and one pharmacist, as well as other general nurses, health assistants, orderlies, and general administrative staff. Three eye care cadres consisting of ophthalmologists, optometrists, and ophthalmic nurses make up the team of prescribers.

### 2.3. Operational Definitions

Acute conjunctivitis: All cases diagnosed as acute conjunctivitis as recorded in the EMR (Medlink software, version 5.7.91) used at the facility.

Appropriate use of antibiotic medication: Antibiotics are used only where there is an indication or suspicion of bacterial infection.

The diagnostic aid provided in the Supplementary Materials (S1 and S2) served as a reference for the classification of the various types of conjunctivitis as agreed upon by the research team at the study centre. For this study, we used the following criteria for ascertaining appropriateness.

1. **Appropriateness**: (a) antibiotics prescribed for acute bacterial conjunctivitis; (b) not prescribing antibiotics in the absence of acute bacterial conjunctivitis.
2. **Inappropriateness**: (a) not prescribing antibiotics when there is acute bacterial conjunctivitis; (b) prescribing antibiotics when there is no indication of bacterial conjunctivitis except where there are extenuating circumstances.

### 2.4. Study Population

The study population consisted of the following:

1. All cases of acute conjunctivitis from 1 January to 31 December 2021 at BAMCEC who were not prescribed antibiotics;
2. All cases of acute conjunctivitis that were reported at BAMCEC from 1 January to 31 December 2024.

### 2.5. Inclusion Criteria

Included in the study were all acute conjunctivitis cases recorded in the Electronic Medical Record (EMR) system (Medlink software) used at the facility (2021 and 2024) and whose treatment cards were available.

### 2.6. Data Variables and Sources of Data

Variables included demographic data, details of diagnosis and treatment, antibiotics prescribed, and the cadre of the prescribeer. These were obtained from the EMR system complemented by information from patient treatment cards.

### 2.7. Data Collection and Entry

For the two time periods, a list of patients in whom the diagnosis was recorded as “acute conjunctivitis” was extracted from the electronic medical records (Medlink software version 5.7.91) as an MS Excel spreadsheet. This spreadsheet included the patient’s folder number (which is a unique identifier for a patient), age, sex, and place of residence.

The patient’s folder number from the list was used to retrieve the patients’ treatment cards. By reviewing the treatment cards, a team of three (two optometrists and one ophthalmic nurse) cross-checked the presenting complaints and symptoms in each folder to ascertain the type of conjunctivitis. The team, guided by the diagnostic aid (see the Supplementary Materials), also extracted other relevant information (whether antibiotics were prescribed, names of antibiotics prescribed, appropriateness of antibiotic prescription or non-prescription) from the patient treatment cards and entered this onto a data collection form created on the Epicollect5 mobile application. Cases of dissenting classification were referred to a senior clinician (deputy chief optometrist) for clarification.

In a situation where further information was missing from the patient treatment card, the case was excluded (34 cases in total).

### 2.8. Data Analysis

The data entered into the Epicollect5 application was downloaded as an MS Excel spreadsheet. This spreadsheet was merged with the MS Excel spreadsheet containing the list of patients from the Medlink software using the VLOOKUP function of MS Excel (Microsoft Corporation, Redmond, WA, USA) with the patient’s folder number as a unique identifier to link the two sheets. This final dataset was imported into STATA version 16 (StataCorp, College Station, TX, USA) for analysis.

Sociodemographic characteristics (age categories, sex, region of residence), types of diagnosis, patterns of antibiotic prescription, and the appropriateness of use or non-use of antibiotics were summarised as numbers and proportions.

To assess the factors associated with antibiotic prescription and appropriateness of antibiotic prescription, multivariable logistic regression was conducted. Unadjusted and adjusted odds ratios (ORs) with 95% confidence intervals (CIs) were reported as measures of association. A *p*-value <0.05 indicated statistical significance.

## 3. Results

### 3.1. Sociodemographic and Clinical Characteristics

There was a total of 3968 conjunctivitis cases recorded from 1 January to 31 December 2024. Of these, 220 (5.5%) had acute conjunctivitis as recorded in the EMR of BAMCEC. There were 119 (54.1%) females and 101 (45.9%) males. The majority of the patients were adults aged eighteen years or over, followed by children under five. Almost all of the patients were from the Central region. Of those diagnosed with AC, 67.3% were prescribed antibiotics—a significantly higher proportion than in 2021 at 55.2% (*p* = 0.011). Details of the sociodemographic and clinical characteristics of patients who visited BAMCEC with acute conjunctivitis in 2024 are provided in Table 1.

### 3.2. Proportions of Patients Prescribed Antibiotics

In unadjusted analyses, those patients who were under 5 years of age, treated in 2024, and from the Central region had higher odds of being prescribed antibiotics.

After adjusting for potential confounders in the multivariable model, year, age and prescriber cadre were significantly associated with the prescription of antibiotics. Those treated in 2024 had three-times-higher odds of being prescribed antibiotics compared to 2021 (aOR 2.51, 95% CI: 1.51–4.19, *p* < 0.001). Patients under five years of age had ten-times-higher odds of being prescribed antibiotics (aOR 10.01, 95% CI: 4.57–21.9, *p* < 0.001). Optometrists were twice as likely to prescribe antibiotics compared to nurses (aOR 2.13, 95% CI: 1.29–3.39). This is shown in Table 2.

**Figure 1:**
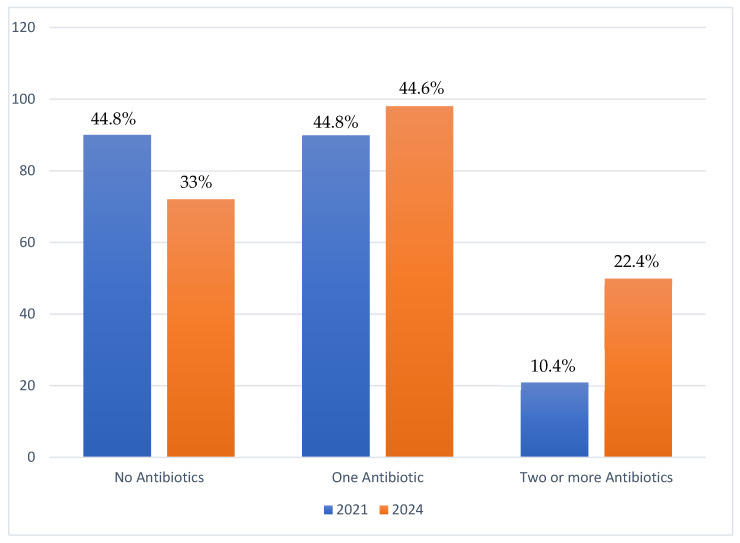
Numbers and proportions of patients receiving antibiotics for acute conjunctivitis at a specialist eye hospital in Ghana in 2021 and 2024.

Of all 220 AC cases, 33% had no antibiotics prescribed, 44.6% had one antibiotic prescribed, and 22.4% had two or more antibiotics prescribed. This contrasts with findings in 2021 where 44.8% of the 201 cases received no antibiotics, while 44.8% received one antibiotic and a further 10.4% receiving more than one antibiotic, as shown in Figure 1.

### 3.3. Appropriateness of Prescribing Antibiotics for Treatment of AC in a Ghanaian Eye Hospital

Out of the total AC cases prescribed antibiotics, the prescription was assessed as “appropriate” in 87.1% (129/148) in 2024, which is a 16% increase compared to the 71% (79/111) reported in 2021 (95% CI for change: 5.99–25.99%, *p* = 0.001).

The appropriateness of antibiotic prescription was further investigated by sex, age-group, and prescribing cadre (ophthalmic nurses or optometrist) for any associations using univariate and multivariate logistic regression (Table 3). On univariate analysis, those patients under five years of age (OR: 2.61, 95% CI: 1.23–5.55) and 18 or over (OR: 3.59, 95% CI: 1.57–8.71) had significantly higher odds of being prescribed antibiotics appropriately compared to those aged 5–17 years. Antibiotic prescriptions in 2024 (OR: 2.75, 95% CI: 1.46–5.18) were significantly more likely to be appropriate. After adjusting for age, gender, region, and prescriber cadre, antibiotic prescriptions of 2024 (aOR: 2.76, 95% CI: 1.41–6.76) were significantly more likely to be appropriate.

### 3.4. Types and Proportions of Antibiotics Prescribed per the WHO AWaRe Classification

**Figure 2:**
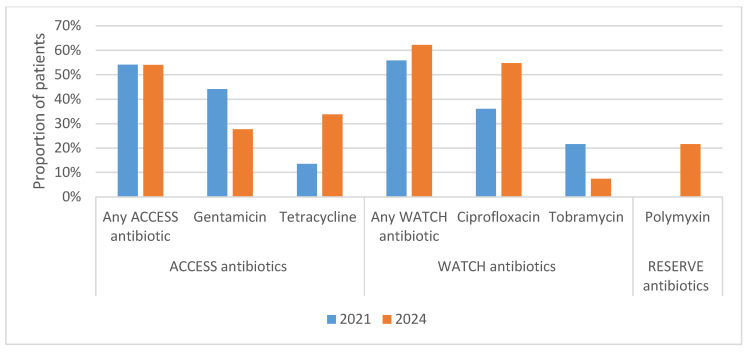
Proportions and distribution of antibiotics prescribed for acute conjunctivitis at a specialist eye hospital in Ghana in 2021 and 2024.

The WHO AWaRe Scheme was used to identify the proportions of prescribed antibiotics in the three categories (ACCESS, WATCH, and RESERVE). Of the total 215 antibiotics prescribed in 2024, 42.3% were in the ACCESS category, 42.8% were in the WATCH category, and 14.9% belonged to the RESERVE category, as represented in Table 4. There was a statistically significant difference between 2021 and 2024 with respect to the RESERVE category (*p* < 0.001) but not in the ACCESS and WATCH categories. The distribution and proportions of ACCESS, WATCH, and RESERVE antibiotics prescribed for AC in 2021 and 2024 are represented in Figure 2.

## 4. Discussion

This research has demonstrated that operational research combined with effective communication of the findings and their implications has the potential to bring about improvements in antibiotic prescribing in eye care.

There were similar numbers of acute conjunctivitis cases in 2021 and 2024 (201 and 220, respectively). The limited availability of data for non-antibiotic cases posed a challenge for comparison. Comparisons were therefore limited to cases where antibiotics were prescribed (111 vs. 148 for 2021 and 2024, respectively).

In the current study, the proportion of AC cases being prescribed antibiotics significantly increased from 55.2% in 2021 to 67.3% in 2024 (*p* = 0.011). The higher proportion of antibiotic prescriptions recorded compared with percentage of AIC is attributable to the treatment of suspected secondary infection and prophylactic treatment in non-infectious acute conjunctivitis. There was also a statistically significant increase in the appropriateness of antibiotic prescription: 87.1% in 2024 compared to 71.2% in 2021 (95% CI for change: 5.99–25.99%, *p* = 0.001). The analysis of the 2021 data on the appropriateness of non-prescription, while limited in terms of the data available, nevertheless indicated that the non-prescription of antibiotics was also highly appropriate and that this had been maintained alongside the improvements in prescribing appropriateness.

Recent findings reveal a shift in the distribution of antibiotic prescription patterns among healthcare cadres. In 2024, optometrists accounted for 71% of prescriptions for acute conjunctivitis, while ophthalmic nurses were responsible for the remaining 29% (*p* = 0.024). This marks a notable change from 2021, when ophthalmic nurses prescribed 46.8% of antibiotics and optometrists or ophthalmologists (predominantly optometrists) accounted for 53.2% [22]. The observed shift in prescribing from ophthalmic nurses to optometrists is attributable to staffing changes during the study period, including the recruitment of two additional optometrists, the placement of an intern optometrist, and the transfer of one ophthalmic nurse. These changes altered the balance of provider encounters and likely influenced the distribution of prescribing roles.

Despite the shift in roles, no significant differences were observed in 2024 between the two cadres in terms of the appropriateness of prescriptions: 88.6% for optometrists and 83.7% for ophthalmic nurses. In comparison, the 2021 proportions were lower (74.6% for optometrists and 67.3% for ophthalmic nurses), indicating improvement in both groups, with ophthalmic nurses showing the most notable progress.

These trends suggest encouraging improvements in prescribing practices across both cadres. However, the observed shift in prescribing roles warrants further investigation to better understand the factors driving this change.

In 2021, there were no statistically significant differences in the appropriateness of antibiotic prescribing across age groups for acute conjunctivitis (AC) cases. However, by 2024, a significant difference was observed. Specifically, 97.6% of children under five years received appropriate antibiotic prescriptions, compared to 70.8% of children aged 5–17 years and 86.8% of adults. This finding is unexplained with the current data and it highlights the need for future training of prescribers to focus on improving antibiotic prescribing practices in specific age groups. Enhancing appropriateness in older children and adults is expected to contribute to improved overall antibiotic stewardship.

The research findings also give some cause for concern and indications for remedial action. While the proportion of AC cases receiving one antibiotic remained comparable (44.8% in 2021 and 44.6% in 2024), the proportion of AC cases being prescribed two or more antibiotics increased from 10.4% in 2021 to 22.7% in 2024. This is indicative of the overuse of antibiotics for a non-lethal condition like AC, a stewardship issue that needs to be addressed through CPD, as recommended by the initial study. More concerning was the emergence of Polymyxin B, a RESERVE category antibiotic, in 14.9% of prescriptions for acute conjunctivitis (AC) in 2024. This marks a significant change, as no RESERVE antibiotics were prescribed for AC during the 2021 study period (*p* < 0.001). The use of Polymyxin B is particularly concerning given that RESERVE antibiotics are intended for use only as a last resort in specific, hard-to-treat infections. A major contributing factor is the use of a fixed-dose combination of Polymyxin B (RESERVE) and tetracycline (ACCESS) as an ophthalmic ointment. Moreover, stock-outs of ACCESS- and WATCH-category antibiotics may have been a possible contributor to this observation. This is only speculative and is not supported by the current data. Nevertheless, these findings remain concerning from a stewardship perspective, as the prescribing of RESERVE antibiotics in combination products for a largely self-limiting condition such as AC is not warranted and requires stricter oversight of ophthalmic prescribing and dosage forms. This highlights the need for robust supply chain management and stricter antibiotic stewardship protocols.

The proportions of ACCESS antibiotics (gentamycin and tetracycline) being prescribed remained similar across the two study periods (46% in 2021, 42.3% in 2024). The proportions of WATCH-category antibiotics (ciprofloxacin and tobramycin) decreased from 54% in 2021 to 42.8% in 2024, which is encouraging. In 2024, the most commonly prescribed antibiotic for AC was ciprofloxacin (36.7% of antibiotic prescriptions), followed by tetracycline (23.3%), gentamycin (19.1%), tobramycin (5.1%), and Polymyxin B (14.9%).

The research and dissemination activities and implemented recommendations may likely be responsible for the improvements in antibiotic prescribing described above. However, incomplete implementation of all recommendations of the initial investigators may have contributed to some of the worrying trends observed. The unimplemented recommendations were as follows:

1. Monitor the use of antibiotics in the eye clinic by the Local Antibiotic Stewardship Team (LAST);
2. Conduct Continuing Professional Development (CPD) training related to antibiotic prescribing through the Drugs and Therapeutic Committee (DTC) at BAMCEC.

The major concerns identified by the research are (1) the rising number of AC cases being prescribed multiple antibiotics, and (2) the inappropriate use of RESERVE-category antibiotics. These negative findings could be attributed to the incomplete implementation of the recommendations. They could have been mitigated if staff had received training through the DTC. Furthermore, even if such prescribing had occurred, LAST could have detected it early enough to ensure more responsible antibiotic use in the eye clinic.

A key strength of this study is its use of routinely collected data involving two full year-long periods (2021 and 2024), providing a large and relatively comprehensive sample of acute conjunctivitis cases that reflects routine prescribing practices. The before–after design allowed changes to be tracked within the same clinical setting, while the integration of antibiotic appropriateness assessments with the WHO AWaRe framework adds global policy relevance. Reporting also adhered to the Strengthening The Reporting of Observational Studies in Epidemiology (STROBE) guidelines [29]. This research contributes to the One Health approach to antimicrobial resistance by highlighting prescribing practices in ophthalmic practice, an area often overlooked in national AMR surveillance. In Ghana, ocular antibiotics are widely used, and inappropriate practices—such as unnecessary prescribing, multiple agents, or RESERVE drug use—pose risks beyond ophthalmic care. Resistant ocular pathogens can spread broadly, adding to the national AMR burden. Our findings therefore complement existing surveillance and emphasise the need to integrate ophthalmic data into Ghana’s One Health AMR stewardship frameworks [17,25,26].

This study is not without its limitations. Changes in medical record systems and practice prevented the retrieval of all 2021 patient records for acute conjunctivitis cases not prescribed antibiotics, as discussed in Section 2. In addition, conjunctivitis classification relied on clinical judgement without microbiological confirmation, and information on disease severity was not captured in the medical records, introducing subjectivity and limiting deeper clinical interpretation. Shifts in the demographic composition of patients between 2021 and 2024 were also observed: for example, the proportion of patients under five fell from 38.3% in 2021 to 20.9% in 2024, while those aged 18 years and above increased from 39.8% to 59.1%; similarly, regional representation shifted markedly, with more cases coming from the Central region in 2024. Such differences may reflect changes in referral pathways, care-seeking behaviour, or service availability and could have influenced prescribing patterns, although regression analyses helped adjust for these variations. Finally, as a retrospective, single-centre study, the findings may not be generalisable to other facilities in Ghana.

## 5. Conclusions

This study has demonstrated that regular operational research coupled with the implementation of associated recommendations has the potential to improve antibiotic prescribing in eye care in Ghana. It allows progress to be monitored and the early detection of worrying practices such as the use of RESERVE antibiotics and prescription of multiple antibiotics for a non-life-threatening condition like AC. It is imperative that stewardship recommendations are fully implemented. Moreover, the study should be replicated in other facilities to include further investigation into the burden, drivers, and clinical outcomes of RESERVE antibiotic use in ophthalmology to inform stewardship policies and enhance AMR surveillance for the protection of the future efficacy of available antibiotics.

## Figures and Tables

**Table 1:** Sociodemographic and clinical characteristics of patients diagnosed with new acute conjunctivitis at a specialist eye hospital in Ghana in 2021 and 2024.

Characteristic	2021 (N = 201)		2024 (N = 220)		*p*-Value ^1^
	*n*	(%)	*n*	(%)	
Age group (years)					
<5	78	(38.8)	46	(20.9)	<0.001
5–17	43	(21.4)	44	(20.0)	
≥18	80	(39.8)	130	(59.1)	
Sex					
Male	89	(44.3)	101	(45.9)	0.737
Female	112	(55.7)	119	(54.1)	
Residence					
Ashanti	0	(0)	2	(0.9)	<0.001
Central	163	(81.1)	212	(96.4)	
Western	32	(15.9)	6	(2.7)	
Western North	5	(2.5)	0	(0)	
Greater Accra	1	(0.5)	0	(0)	
Prescriber cadre					
Ophthalmologist	2	(1.0)	0	(0)	0.008
Optometrist	107	(53.2)	145	(65.9)	
Ophthalmic nurse	92	(45.8)	75	(34.1)	
Type of acute conjunctivitis					
Bacterial	NA		89	(40.4)	-
Viral	NA		20	(9.1)	
Non-infectious	NA		111	(50.5)	
Prescribed antibiotics					
Yes	111	(55.2)	148	(67.3)	0.011
No	90	(44.8)	72	(32.7)	

NA—Data not available. ^1^ z-test.

**Table 2:** Logistic regression analysis of demographic and prescriber characteristics associated with antibiotic prescription in new acute conjunctivitis among patients who were prescribed antibiotics at a specialist eye hospital in Ghana in 2021 and 2024.

Variable	Total	Antibiotic Prescription		OR	(95% CI)	aOR	(95% CI)	*p*-Value
		*n*	(%) ^1^					
Year								
2021	201	111	(55.2)	Ref		Ref		
2024	220	148	(67.3)	1.67	(1.12–2.47)	2.51	(1.51–4.19)	<0.001
Age (years)								
<5	124	113	(91.1)	7.60	(3.58–16.10)	10.01	(4.57–21.9)	<0.001
5–17	87	50	(57.5)	Ref		Ref		
≥18	210	96	(45.7)	0.62	(0.37–1.03)	0.52	(0.29–0.93)	0.027
Sex								
Male	190	117	(61.6)	Ref		Ref		
Female	231	142	(61.5)	0.99	(0.67–1.48)	1.16	(072–1.86)	0.525
Region								
Central	375	248	(66.1)	6.29	(2.89–13.7)	2.26	(0.95–5.41)	0.067
Western	38	9	(23.7)	Ref		Ref		
Other regions	8	2	(25.0)	1.07	(0.18–6.28)	0.59	(0.07–5.01)	0.634
Prescriber cadre								
Optometrist	254	164	(64.6)	1.38	(0.92–2.06)	2.13	(1.29–3.39)	0.003
Ophthalmic nurse	167	95	(56.9)	Ref		Ref		

^1^ Row percentages, calculated from the total number in each group. Abbreviations: OR: unadjusted odds ratio; aOR: adjusted odds ratio; CI: confidence interval.

**Table 3:** Logistic regression analysis of demographic and prescriber characteristics associated with appropriateness of antibiotic prescription for new acute conjunctivitis in patients who were prescribed antibiotics at a specialist eye hospital in Ghana in 2021 and 2024.

Variable	Total	Appropriate Antibiotic Prescription		OR	(95% CI)	aOR	(95% CI)	*p*-Value
		*n*	(%) ^1^					
Year								
2021	111	79	(71.2)	Ref		Ref		
2024	148	129	(87.2)	2.75	(1.46–5.18)	2.76	(1.32–5.78)	0.007
Age (years)								
<5	113	93	(82.3)	2.61	(1.23–5.55)	3.04	(1.41–6.76)	0.005
5–17	50	32	(64.0)	Ref		Ref		
≥18	96	83	(86.5)	3.59	(1.57–8.17)	2.22	(0.87–5.67)	0.093
Sex								
Male	117	92	(78.6)	Ref		Ref		
Female	142	116	(81.7)	1.21	(0.65–2.24)	1.07	(0.55–2.06)	0.833
Region								
Central	248	199	(80.2)	1.16	(0.23–5.76)	1.18	(0.21–6.69)	0.846
Western	9	7	(77.8)	Ref		Ref		
Other regions	2	2	(100.0)	-		-		
Prescriber cadre								
Optometrist	164	137	(83.5)	1.71	(0.92–3.19)	1.43	(0.73–2.80)	0.297
Ophthalmic nurse	95	71	(74.7)	Ref		Ref		

^1^ Row percentages, calculated from the total number in each group. Abbreviations: OR: unadjusted odds ratio; aOR: adjusted odds ratio; CI: confidence interval.

**Table 4:** Types of antibiotics prescribed as per the WHO AWaRe classification of antibiotics in patients diagnosed with new acute conjunctivitis at a specialist eye hospital in Ghana in 2021 and 2024.

	2021 (N = 131)		2024 (N = 215)		*p*-Value ^2^
	*n*	(%) ^1^	*n*	(%) ^1^	
Access antibiotics	64	(48.8)	91	(42.3)	<0.001
Watch antibiotics	67	(51.2)	92	(42.8)	
Reserve antibiotics	0	(0)	32	(14.9)	

^1^ Percentages calculated from the total number of antibiotics prescribed as the denominator. ^2^ Pearson’s Chi-square test.

## Data Availability

The datasets presented in this article are not readily available due to confidentiality and ethical restrictions. Requests to access these data should be sent to the corresponding author.

## Supplementary Materials

The following supporting information can be downloaded at https://www.mdpi.com/article/10.3390/tropicalmed10110301/s1, Supplement S1. Classification of conjunctivitis; Supplement S2. Symptom Criteria for acute conjunctivitis; Supplement S3. Dissemination details of the baseline operational research study; Supplement S4. Recommendations, action status, and details of actions from the baseline study.
