# Supplementary material for: Improved Antibiotic Prescribing for Acute Conjunctivitis After Operational Research: A Before-and-After Study in a Ghanaian Eye Hospital
Source: Trop Med Infect Dis. 2025 Oct 22;10(11):301. doi: 10.3390/tropicalmed10110301 (PMC12656491; doi:10.3390/tropicalmed10110301)
Supplement: Supplementary file 1 [file tropicalmed-10-00301-s001.zip › tropicalmed-3865721-supplementary.pdf]

Supplementary Materials:

Supplement S1. Classification of conjunctivitis. Adapted from Standard Treatment Guidelines 7th edition [11]

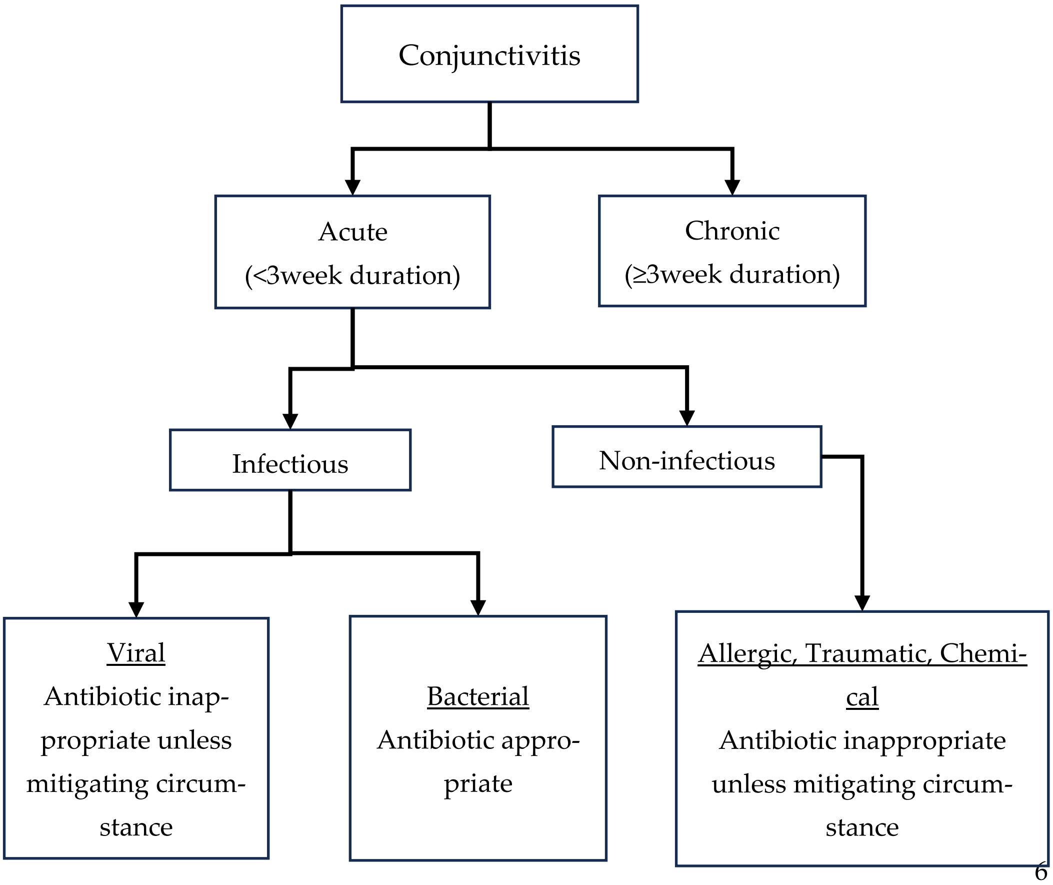

Supplement S2.

Symptom Criteria for acute conjunctivitis. Adapted from 7th Edition of the Ghana Standard Treatment Guidelines 2017 (STG) [11]

**Symptoms**	Allergic conjunctivitis	Bacterial conjunctivitis	Viral conjunctivitis
**Appearance of discharge**	White stringy mucoid	Mucopurulent	Watery
**Presence of erythema**	Mild to moderate	Moderate to severe	Mild to moderate
**Pruritus**	Moderate to severe	None to mild	Mild to moderate
**Bilateral eye involvement**	Common	Unilateral initially	Rare
**Presence of lymphadenopathy**	None	Rare	Common
**Upper respiratory coinfection**	None	Rare	Common

Supplement S3. Dissemination details of the baseline operational research study

How	To Whom	Where (Number)	When
1st Engagement with management of BAMCEC	Management Team of BAMCEC	Clinic premises (6)	November 25, 2022
SORT IT Dissemination	National Stakeholders	Oak Plaza Hotel, Accra.	July 05, 2023
Discussion	Head of Ghana Eye Care Secretariat.	Oak Plaza Hotel, Accra. (1)	July 05, 2023
2nd Engagement with management of BAMCEC	Management Team of BAMCEC	Clinic premises (7)	July 28, 2023
Engagement with prescribers and pharmacist of BAMCEC	Prescribers and pharmacist of BAMCEC	Clinic premises (10)	August 02, 2023

Supplement S4. Recommendations, action status, and details of actions from the baseline study

Recommendation	Action Status	Details of Action (When and What)
Brief Management of BAMCEC on outcome and implications of study	Implemented	November 2022<br>Details of inappropriate use of antibiotics were shared
Brief Head of Ghana Eye Care Secretariat on outcome and implications of study.	Implemented	July 2023<br>Details of inappropriate use of antibiotics were shared
Brief BAMCEC prescribers on outcome and implications of study.	Implemented	August 2023<br>Details of inappropriate use of antibiotics were shared
Establish Local Antibiotic Stewardship Team (LAST)	Implemented	July 2023<br>Management has formed local antibiotic stewardship team
Monitoring use of antibiotics in the Eye Clinic by LAST	Not done	
Running of CPDs by Drugs and Therapeutic Committee	Not done	
Replication of study by other eye care facilities spearheaded by the Ghana Eye Care Secretariat	Not done	
